# Defining laboratory medicine: a circle cannot be squared

**DOI:** 10.11613/BM.2021.020401

**Published:** 2021-06-15

**Authors:** Giuseppe Lippi, Mario Plebani

**Affiliations:** 1 Section of Clinical Biochemistry, University of Verona, Verona, Italy; 2 Department of Laboratory Medicine, University Hospital of Padova, Padova, Italy

**Keywords:** laboratory medicine, testing, definition

## Abstract

At the down of the third millennium, it is rather misleading to consider the “whole population” as a conceptual entity, whereby the population is actually composed by single individuals, who differ broadly in terms of age, sex, ethnic origin, occupation, health, wellbeing, lifestyle and risk factors. While reaffirming strongly that laboratory medicine shall aim to provide data that could be translated into actionable information on “BOTH” an individual and universal level, we confute and refuse the naive and too simplistic approach that the common beneficence shall always be prioritized over the individual good, since the common good is just the sum of many individual beneficences.

## To the Editor,

We do acknowledge the comment of Joseph Watine on our speculative definition of laboratory medicine, earlier published in the journal *Clinical Chemistry and Laboratory Medicine* (*1*, *2*). Although we would like to emphasize first that it does not appear a conventional nor appropriate practice to publish rebuttals in journals other than those where the original manuscript has been published, particularly because this does not easily allow a reply by the authors, we feel almost unavoidable to provide a comment on Watine’s correspondence (*2*).

There is perhaps a general misconception in the use that Watine does of our definition of laboratory medicine, since in no way we aimed to prioritize the benefit (or advantage) of the single individual versus that of the community. Rather, we reinforce our conviction that care improvements and/or wellness maintenance are processes that shall consider “BOTH” the single individual as well as the entire population. At the down of the third millennium, it is rather misleading to consider the “whole population” as a conceptual entity, whereby the population is actually composed by single individuals, who differ broadly in terms of age, sex, ethnic origin, occupation, health, wellbeing, lifestyle and risk factors. Watine seems to ignore the foremost importance of personalized (laboratory) medicine, where diagnosis and treatment need now to be tailored according to specific genetic, phenotypic and even environmental factors (*3*). Even more accountably, Watine seems to overlook that what seems to work in a population with specific characteristics does not necessary translate its benefits in the single individuals or in other (different) cohorts. Cancer diagnostic is a paradigmatic example, whereby dissection of complex molecular and/or biochemical signatures in the individual patient has now become possible with “omics” technologies, encompassing high throughput instrumentation combined with artificial intelligence (*4*). This approach would enable to provide objective benefits to a single individual, which would then translate into paramount clinical, societal and even economical revenues. Pragmatically, and bringing a current tragedy as an example, the diagnosis of coronavirus disease 2019 (COVID-19) would carry many drawbacks adapting a universal diagnostic strategy, since the diagnosis of severe acute respiratory syndrome coronavirus 2 (SARS-CoV-2) infection benefits from multiple testing according to the “precise” diseased state of the patient (*i.e.*, nasopharyngeal testing in symptomatic individuals, saliva testing in those with asymptomatic disease, analysis of bronchoalveolar lavage or stool in patients with long persistence of systemic disease, and so forth) (*5*). These are just two common examples of how adopting a biased view towards the “beneficence of the majority” would generate many clinical, diagnostic and even ethical drawbacks in the care of the “isolate subject”.

In conclusion, while reaffirming strongly that laboratory medicine shall aim to provide data that could be translated into actionable information on “BOTH” an individual and universal level, we confute and refuse the naive and too simplistic approach suggested by Joseph Watine that the common beneficence shall always be prioritized over the individual good, since the common good is just the sum of many individual beneficences.
